# Genomic analysis of KEL*03 and KEL*04 alleles among Thai blood donors

**DOI:** 10.4102/ajlm.v13i1.2294

**Published:** 2024-03-19

**Authors:** Oytip Nathalang, Panasya Rassuree, Kamphon Intharanut, Wanlapa Chaibangyang, Núria Nogués

**Affiliations:** 1 Graduate Program in Medical Technology, Faculty of Allied Health Sciences, Thammasat University, Pathumthani, Thailand; 2 Laboratori d’Immunohematologia Banc de Sang i Teixits, Barcelona, Spain

**Keywords:** Kell blood group system, phenotype, allele, genotype, Thai population

## Abstract

**Background:**

The Kell blood group system is clinically important in transfusion medicine, particularly in patients with antibodies specific to Kell antigens. To date, genetic variations of the Kell metallo-endopeptidase (*KEL*) gene among Thai populations remain unknown.

**Objective:**

This study aimed to determine the frequencies of KEL*03 and KEL*04 alleles among Thai blood donors using an in-house polymerase chain reaction-sequence-specific primer (PCR-SSP) method.

**Methods:**

Blood samples obtained from 805 unrelated central Thai blood donors at a blood bank in Pathumthani, Thailand, from March 2023 to June 2023, were typed for Kp^a^ and Kp^b^ antigens using the column agglutination test, and the results for 400 samples were confirmed using DNA sequencing. A PCR-SSP method was developed to detect the KEL*03 and KEL*04 alleles, and genotyping results were validated using known DNA controls. DNA samples obtained from Thai donors in central (*n* = 2529), northern (*n* = 300), and southern (*n* = 427) Thailand were also genotyped using PCR-SSP for comparison.

**Results:**

All 805 (100%) donors had the Kp(a−b+) phenotype. The PCR-SSP genotyping results agreed with the column agglutination test and DNA sequencing. All 3256 Thai blood donors had the homozygous KEL*04/KEL*04 genotype. Frequencies of the KEL*03 and KEL*04 alleles among Thai donors differed significantly from those of Japanese, Native American, South African, Brazilian, Swiss, and German populations.

**Conclusion:**

This study found a 100% KEL*04 allele frequency in three Thai populations. These data could provide information on KEL*03 and KEL*04 allele frequencies to estimate the risk of alloimmunisation in Thai populations.

**What this study adds:**

This study demonstrates that in-house PCR-SSP can be used to determine KEL*03 and KEL*04 alleles to predict Kp^a^ and Kp^b^ antigens. Even though only homozygous KEL*04/KEL*04 genotypes were found among Thai donor populations, the established PCR-SSP method may be useful for estimating the risk of alloimmunisation in other populations.

## Introduction

The high immunogenicity of antigens in the Kell blood group system (International Society of Blood Transfusion symbol, KEL, and number, 006), particularly the K antigen, is clinically significant in transfusion medicine.^1^ Kell antigens are detectable as early as the 10th week of pregnancy and reach full maturity after birth. Immunoglobulin G antibodies against Kell antigens may develop during transfusion or pregnancy, leading to haemolytic transfusion reactions and haemolytic disease of the foetus and newborn.^2,3^ Currently, 37 antigens have been identified in this system. Individuals without Kell antigens are referred to as having the K_0_ phenotype (Kell null), which is very rare across all populations. Patients with antibodies against Kell antigens, such as K/k (KEL1/KEL2), Kp^a^/Kp^b^ (KEL3/KEL4) and Js^a^/Js^b^ (KEL6/KEL7), frequently experience haemolytic transfusion reactions and haemolytic disease of the foetus and newborn issues.^2,4^

In general, Kell antigen frequencies differ among populations. For example, the frequency of the K+ phenotype is high among Caucasians (7% – 9%) but less than 0.1% among Asians.^1,3^ In a related study of 1522 Thai blood donors from the National Blood Centre, Thai Red Cross Society, the K−k+ phenotype was the most common (98.16%), followed by K+k+ (1.78%) and K+k− (0.07%).^4^ Low-incidence antigens like the Kp^a^ antigen are present in less than 1% of the general population, whereas high-incidence antigens like the Kp^b^ antigen are found in more than 99% of the population.^1,2,3^ Kp^a^ and Kp^b^ antigens are not included in screening or panel cells. Hence, additional panel cells are needed to identify antibody specificity among patients with antibodies against these antigens (anti-Kp^a^ or anti-Kp^b^).^2,5^

Anti-Kp^a^ and anti-Kp^b^ are typically Immunoglobulin G antibodies and exhibit reactivity in the indirect antiglobulin test, especially when using enzyme-treated red blood cells. The conventional tube test and column agglutination test (CAT) are routinely used to identify Kp^a^ and Kp^b^ antigens and to determine Kp(a+b−), Kp(a+b+), and Kp(a−b+) phenotypes.^1,6^ The limitations of typing for these antigens using conventional tube test and CAT methods include the high cost of commercial antiserum, as well as the observation of positive direct antiglobulin test and positive autocontrol results in chronically transfused patients.^1,2,3^ Genotyping of the Kell metallo-endopeptidase (*KEL*) gene for the KEL*03 and KEL*04 alleles is an alternative to serological testing for predicting Kp^a^ and Kp^b^ antigens. It is useful for selecting appropriate blood for patients with anti-Kp^a^ and anti-Kp^b^ antibodies, as well as in anthropology studies to determine allele frequencies within populations.^7,8,9^

Various polymerase chain reaction (PCR)-based techniques have been used to identify the KEL*03 and KEL*04 alleles. The PCR-restriction fragment length polymorphism and polymerase chain reaction-sequence-specific primer (PCR-SSP) methods have been used to genotype these alleles among Iranian patients with alloimmunised multi-transfused thalassaemia^7,8^ and to determine allele frequencies in the multi-ethnic Brazilian population.^9^ A multiplex PCR assay was also developed to genotype 35 red blood cell antigens among Austrian blood donors and provided reliable results compared with serological testing.^10^ Subsequently, Luminex-based genotyping platforms have also been implemented to predict red blood cell phenotypes among patient and donor populations.^11,12,13,14,15^ Furthermore, matrix-assisted laser desorption/ionisation time of flight mass spectrometry was used to determine blood group alleles in blood donors.^16,17,18^ This method offered high throughput and proved to be less time-consuming than routine serological testing.^16,17,18^ However, genotyping using Luminex-based platforms and matrix-assisted laser desorption/ionisation time of flight mass spectrometry techniques requires costly reagents and equipment, thus making them unsuitable for laboratories with established budgets. Since the KEL*03 and KEL*04 allele frequencies in Thai populations remain unknown, this study aimed to detect KEL*03 and KEL*04 alleles among blood donors in central, northern, and southern Thailand using in-house PCR-SSP and to compare the observed allele frequencies with those of other donor populations.

## Methods

### Ethical considerations

Ethical clearance to conduct this study was obtained from the Human Research Ethics Committee of Thammasat University, Thailand (No. COE No. 007/2566), according to the Declaration of Helsinki. Written informed consent was obtained from all individuals.

### Study population

Leftover ethylenediaminetetraacetic acid-anticoagulated blood samples were collected from 805 unrelated healthy Thai blood donors at the Blood Bank, Thammasat University Hospital, Pathumthani, Thailand, from March 2023 to June 2023. All the donors who qualified for inclusion had already passed the questionnaire and physical screening procedures for blood donation provided by the National Blood Centre, Thai Red Cross Society. Blood samples positive for hepatitis B virus, hepatitis C virus, HIV, or syphilis were excluded from the study. In addition, the study included 1724 leftover DNA samples from the Blood Bank, Thammasat University Hospital, Pathumthani; 300 samples from the Blood Bank Section, Faculty of Medicine, Chiang Mai University, Chiang Mai; and 427 samples from the Regional Blood Centre, 12th Songkhla, Thai Red Cross Society, Songkhla, Thailand. DNA controls for the Kp(a+b−) and Kp(a+b+) phenotypes were obtained from Laboratori d’Immunohematologia Banc de Sang i Teixits, Barcelona, Spain.

### DNA extraction

Genomic DNA was extracted from the samples using the DNeasy Blood & Tissue Kit (QIAGEN, Valencia, California, United States) according to the manufacturer’s instructions and stored at −20 °C before further analysis.

### Serological testing for Kp^a^ and Kp^b^ antigen typing

Altogether, 805 blood samples were analysed to detect the Kp^a^ and Kp^b^ antigens, using the CAT method with an anti-Kp^a^/Kp^b^ ID-Card (Bio-Rad Laboratories, Cressier, Switzerland) made up of six microtubes containing either anti-Kp^a^ or anti-Kp^b^ of human origin within the gel matrix. Briefly, 10 µL of 5% red cell suspension in ID-Diluent 1 (Bio-Rad Laboratories, Cressier, Switzerland) was added to the appropriate microtubes of the ID-Card. The ID Card was then centrifuged, and the results were recorded.

### Sequence-based genotyping

Genomic DNA from 400 of the samples tested using CAT were randomly selected and sequenced to identify KEL*03 and KEL*04 alleles. Primers were designed for each target gene (Table 1) using National Center for Biotechnology Information Primer-BLAST (https://www.ncbi.nlm.nih.gov/tools/primer-blast/).^19^ Polymerase chain reaction amplification for the *KEL* gene exon 8 containing single nucleotide variant c.841 C > T [rs8176059], p.Arg281Trp, was performed using SEQ-KP-F forward and SEQ-KP-R reverse primers, with an expected fragment size of 358 base pairs (bp). Each PCR reaction mix (total volume: 40 µL) was prepared using 3 µL of genomic DNA (50 ng/L), 3 µL each of the forward and reverse primers (10 µM), 20 µL of 2X PCR reaction mixture (Phusion High-Fidelity PCR Master Mix, New England BioLabs, Ipswich, Massachusetts, United States), and 11 µL of sterile distilled water. The PCR reactions were carried out in a T100 Thermal Cycler (Bio-Rad Laboratories, Inc., Hercules, California, United States). PCR reactions included 30 s of initial denaturation at 98 °C, followed by 10 cycles of 10 s at 98 °C and 60 s at 69 °C, then 20 cycles of 10 s at 98 °C, 50 s at 60 °C, and 30 s at 72 °C, and a final extension step for 5 min at 72 °C. The PCR products were separated on a 1.5% agarose gel with a 100 bp DNA molecular weight marker (Fermentas, Carlsbad, California, United States), stained using SYBR Safe DNA Gel Stain (Invitrogen, Paisley, United Kingdom), electrophoresed in 1X Tris-borate-EDTA solution at 100 Volts for 30 min, and then visualised with a blue-light transilluminator. The PCR products were then purified using a gel extraction kit (GeneJet Gel Extraction Kit, Fisher Scientific Baltics UAB, Vilnius, Lithuania), and the eluted fragments were sequenced directly using Sanger sequencing (U2Bio Sequencing Service, Bangkok, Thailand) with the aforementioned PCR primers.

**TABLE 1:** Primer sequences designed for the detection of KEL*03 and KEL*04 alleles from samples of healthy Thai blood donors at Thammasat University Hospital, Thailand, March 2023 – June 2023.

Target	Primers	Primer sequence (5′-3′)	Product size (bp)	Uses
*KEL* gene	SE-KP-F	AACAGAAGATCTATGCCCAGG	358	PCR amplification, Sequencing
	SE-KP-R	AATGTTTGAGAGGAAGATCCCCA		
KEL*03 allele	KP-t-A-F	CCTTGTCAATCTCCATCACTTCAT	158	PCR-SSP
	KP-REV	TGAGAGGAAGATCCCCATGCC		
KEL*04 allele	KP-c-B-F	CCTTGTCAATCTCCATCACTTCAC	158	PCR-SSP
	KP-REV	TGAGAGGAAGATCCCCATGCC		
*HGH* gene	HGH-F	TGCCTTCCCAACCATTCCCTTA	434	PCR-SSP
	HGH-R	CCACTCACGGATTTCTGTTGTGTTTC		

*KEL*, Kell metallo-endopeptidase gene; *HGH*, human growth hormone; PCR, polymerase chain reaction; PCR-SSP, PCR sequence-specific primer; bp, base pair.

### Genotyping using polymerase chain reaction-sequence-specific primer

KEL*03 and KEL*04 alleles were identified using an in-house PCR-SSP technique with custom primers and amplification conditions (Table 1). The KP-t-A-F and KP-REV primers were used for KEL*03 amplification, while the KP-c-B-F and KP-REV primers were used for KEL*04 amplification. Two sets of PCR reaction mixtures, set A for identifying KEL*03 alleles and set B for KEL*04 alleles, were performed for each sample. Each PCR reaction mixture (total volume: 10 µL) was prepared using 1 µL of template genomic DNA (50 ng/L), 1 µL each of the forward and reverse primers (10 µM), and 5 µL of 2 × PCR reaction mixture (Green Hot Start PCR Master Mix, Biotechrabbit GmbH, Hennigsdorf, Germany). Co-amplification of the human growth hormone (*HGH*) gene using 1 µL of HGH-F and HGH-R primers (6 µM) was conducted as the internal control in each set. The PCR reactions were carried out in a T100 Thermal Cycler (Bio-Rad Laboratories, Inc., Hercules, California, United States) and included 30 s of initial denaturation at 95 °C, followed by 10 cycles of 30 s at 95 °C and 60 s at 69 °C, then 20 cycles of 10 s at 95 °C, 50 s at 62 °C and 30 s at 72 °C, and a final extension step for 5 min at 72 °C. After electrophoresis, PCR products were examined with a blue-light transilluminator. The expected PCR product size for both the KEL*03 and KEL*04 amplification reactions was 158 bp, while that of the internal control, the *HGH* gene, was 434 bp. Known DNA controls of KEL*03/KEL*03, KEL*03/KEL*04 and KEL*04/KEL*04 genotypes were used as positive and negative controls in parallel with the test samples. The KEL*03/KEL*03 genotype was indicated if amplification was observed with only the KEL*03 reaction (set A), while the KEL*04/KEL*04 genotype was indicated if amplification was observed with only the KEL*04 reaction (set B). If amplification occurred in both sets of reactions, the KEL*03/KEL*04 genotype was indicated. The specificity of PCR-SSP was examined by cross-amplifying the KEL*03-specific primers with the KEL*04/KEL*04 genotype and vice versa. The PCR-SSP method was conducted on a total of 3256 DNA samples (including the 400 samples tested using DNA sequencing) to identify KEL*03 and KEL*04 alleles.

### Repeatability

To evaluate the repeatability of the newly developed PCR-SSP, 100 randomly selected samples with known genotypes by DNA sequencing were retested by PCR-SSP. Upon completion of PCR-SSP genotyping in all 3256 samples, 100 randomly selected DNA samples from among the 400 blood donors with genotyping obtained by standard DNA sequencing were retested using PCR-SSP under the same conditions as previously described to test the repeatability of our in-house PCR-SSP technique for KEL*03 and KEL*04 genotyping. To improve the validity and reliability of the evaluation, technicians were blinded to the PCR-SSP results. DNA sequencing was performed in the event of any discrepant genotyping results obtained using the PCR-SSP and serological assays.

### Comparison of KEL*03 and KEL*04 allele frequencies between populations

KEL*03 and KEL*04 allele frequencies among Thai blood donors were compared with those reported in Filipino, Southeast Asian, Chinese, Japanese, Korean, South Asian, Native American, Alaska Native/Aleut, Hawaiian/Pacific Islander, South African, Brazilian, Swiss, and German populations.^9,11,15,16,17^

### Statistical analysis

Gene and allele frequencies were simply estimated by counting the number of times each gene and allele was observed in samples from each population. The calculation of allele frequency for KEL*03 is based on the gene counting method; every KEL*03/KEL*03 individual has two KEL*03 alleles, and a KEL*03/KEL*04 individual has one. This is relative to all alleles in the population, that is, two times the total number of samples. This was also applied to the KEL*04 allele frequency calculation:

$$\begin{array}{l} \mathrm{KEL}*03\text{ allele frequency}=(2\times \mathrm{KEL}*03/\mathrm{KEL}*03+\\ \mathrm{KEL}*03/\mathrm{KEL}*04)/(2\times \text{the total number of samples}) \end{array}$$ [Eqn 1]

$$\begin{array}{l} \mathrm{KEL}*04\text{ allele frequency}=(2\times \mathrm{KEL}*04/\mathrm{KEL}*04+\\ \mathrm{KEL}*03/\mathrm{KEL}*04)/(2\times \text{the total number of samples}) \end{array}$$ [Eqn 2]

The Chi-square (χ^2^) test was used to evaluate whether the observed genotype frequencies agreed with the expected frequencies under the Hardy-Weinberg equilibrium. The two-tailed Fisher’s exact test with Bonferroni adjustment of homogeneity was used to determine the differences in allele frequencies between central, northern, and southern Thai populations and to compare these differences with those reported in other populations.^9,11,15,16,17^ An adjusted *p*-value of less than 0.004 was considered statistically significant. All statistical analyses were conducted using SPSS^®^ version 16.0 (SPSS Inc., Chicago, Illinois, United States).

## Results

### Serological testing and DNA sequencing

**FIGURE 1:**
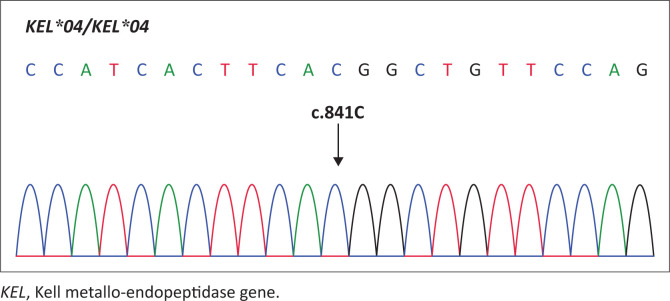
Electropherogram of the sequenced KEL*03 and KEL*04 polymorphism region of the *KEL* gene in a Thai blood donor, Thammasat University Hospital, Thailand, March 2023 – June 2023. The homozygous state of the c.841C position at exon 8 identified in KEL*04/KEL*04 genotypes is shown.

A total of 805 blood samples were tested for Kp^a^ and Kp^b^ antigens using CAT. All donors (100%) had the Kp(a−b+) phenotype. Kp(a+b−) and Kp(a+b+) phenotypes were not found in this donor population. The sequencing results of the 400 randomly selected samples revealed that these donors were homozygous at the *KEL* gene locus, having the KEL*04/KEL*04 genotype (Figure 1). These results agreed with the serological testing results using CAT.

### Genotyping using polymerase chain reaction-sequence-specific primer

**FIGURE 2:**
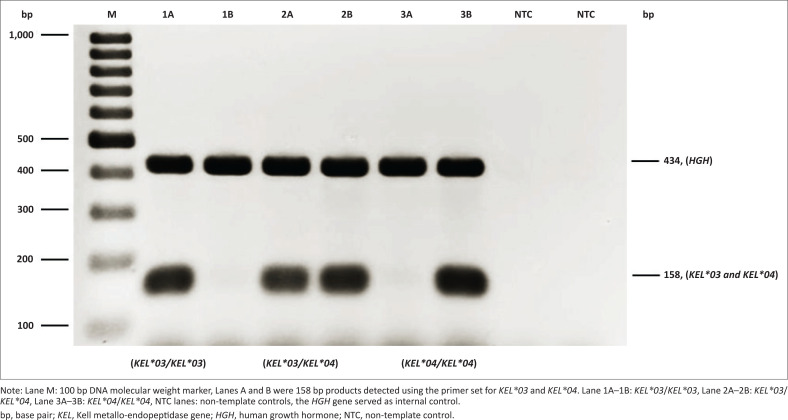
Genotyping of KEL*03 and KEL*04 alleles using known DNA controls by polymerase chain reaction sequence-specific primers at Thammasat University Hospital, Thailand, March 2023 – June 2023.

According to the PCR-SSP results, among 3256 examined samples, a 158 bp product was only detected using the particular primer sets for KEL*04 allele detection, thus indicative of the KEL*04/KEL*04 genotype. The *HGH* internal control was also amplified in all reactions (Figure 2). Three samples with the Kp(a−b+), Kp(a+b−), and Kp(a+b+) phenotypes as determined using DNA sequencing were used as known DNA controls to validate the genotyping results for both alleles. For all three control samples, there was no cross-amplification detected between KEL*03 and KEL*04 primer sets.

In total, to detect KEL*03 and KEL*04 alleles, we genotyped 2529 samples obtained from central Thai blood donors, 300 samples from northern Thai donors, and 427 samples from southern Thai donors using the newly developed PCR-SSP. All 3256 Thai blood donors were homozygous at the *KEL* gene locus, having the KEL*04/KEL*04 genotype. The KEL*03/KEL*03 and KEL*03/KEL*04 genotypes were not detected (Table 2). The retesting results for the sub-group of 100 blood sample donors with known genotype agreed with the first round of PCR-SSP testing for all samples. The frequencies of the three genotypes, including KEL*03/KEL*03, KEL*03/KEL*04, and KEL*04/KEL*04 genotypes, were consistent between the central Thai, northern Thai, and southern Thai populations according to the Hardy-Weinberg equilibrium (χ^2^ = 0.00, degree of freedom = 2, *p* = 1.00).

**TABLE 2:** KEL*03 and KEL*04 genotype and allele frequencies among Thai blood donors at Thammasat University Hospital, Thailand, March 2023 – June 2023.

*KEL* gene variants	Population						Total (*N* = 3256)	
	Central Thai (*n* = 2529)		Northern Thai (*n* = 300)		Southern Thai (*n* = 427)			
	*n*	%	*n*	%	*n*	%	*n*	%
**Allele†**								
KEL * 03	0	0.00	0	0.00	0	0.00	0	0.00
KEL * 04	5058	100.00	600	100.00	854	100.00	6512	100.00
**Genotype**								
KEL*03/KEL*03	0	0.00	0	0.00	0	0.00	0	0.00
KEL*03/KEL*04	0	0.00	0	0.00	0	0.00	0	0.00
KEL*04/KEL*04	2529	100.00	300	100.00	427	100.00	3256	100.00

† , Allele frequencies are reported as two times the total number of samples (each individual has two copies).

*KEL*, Kell metallo-endopeptidase gene.

### KEL*03 and KEL*04 allele frequencies in Thai and other populations

The KEL*03 and KEL*04 frequencies among the Thai donors in this study were similar to those reported in Filipino (*p* = 0.29),^11^ Southeast Asian (*p* = 1.00),^11^ Chinese (*p* = 0.12),^11^ Korean (*p* = 1.00),^11^ South Asian (*p* = 0.05),^11^ Alaska Native/Aleut (*p* = 0.008),^11^ and Hawaiian/Pacific Islander (*p* = 1.00) populations^11^ (Table 3). There were, however, significant differences in KEL*03 and KEL*04 allele frequencies between the Thai donors in this study and individuals in a South African population^15^ (KEL*03: 0.0000 vs 0.0067; and KEL*04: 1.0000 vs 0.9933; *p* = 0.002). In addition, there were significant differences in allele frequencies between Thai donors and individuals in Japanese (*p* < 0.001),^11^ Native American (*p* < 0.001),^11^ Brazilian (*p* < 0.001),^9^ Swiss (*p* < 0.001),^16^ and German populations (*p* < 0.001).^17^

**TABLE 3:** Comparison of the KEL*03 and KEL*04 allele frequencies between Thai blood donors (Thammasat University Hospital, Thailand, March 2023 – June 2023) and blood donors from other populations.

Population	Number of samples	Number of alleles				Adjusted *p*-value	Allele detection method used
		KEL*03		KEL*04			
		*n*	%	*n*	%		
Thai (this study)	3256	0	0.00	3256	100.00	-	PCR-SSP
Filipino^11^	1333	1	0.05	2665	99.95	0.291	DNA array
Southeast Asian^11^	942	0	0.00	942	100.00	1.000	DNA array
Chinese^11^	1715	2	0.05	3428	99.95	0.119	DNA array
Japanese^11^	1022	14	0.70	2030	99.30	< 0.001*	DNA array
Korean^11^	1033	0	0.00	1033	100.00	1.000	DNA array
South Asian^11^	922	2	0.10	1842	99.90	0.049	DNA array
Native American^11^	485	6	0.65	964	99.35	< 0.001*	DNA array
Alaska Native/Aleut^11^	311	2	0.40	620	99.60	0.008	DNA array
Hawaiian/Pacific Islander^11^	261	0	0.00	261	100.00	1.000	DNA array
South African^15^	150	2	0.67	298	99.33	0.002*	Luminex-based assay
Brazilian^9^	800	11	0.69	1589	99.31	< 0.001*	PCR-RFLP
Swiss^16^	4000	108	1.35	7892	98.65	< 0.001*	MALDI-TOF MS
German^17^	20 529	328	0.80	40 730	99.20	< 0.001*	MALDI-TOF MS

*KEL*, Kell metallo-endopeptidase gene; MALDI-TOF MS, matrix-assisted laser desorption/ionisation time of flight mass spectrometry; PCR-RFLP, polymerase chain reaction restriction fragment length polymorphism; PCR-SSP, PCR sequence-specific primer.

* , Adjusted *p* < 0.004 (Bonferroni adjustment).

## Discussion

In this study, 3256 DNA samples from three Thai blood donor populations were tested using PCR-SSP. The KEL*03 allele predicting the Kp^a^ antigen was not observed. Among the 805 samples from central Thai blood donors tested using CAT in this study, the Kp(a−b+) phenotype was the only phenotype detected (100%). The sequencing results of 400 of these 805 samples confirmed the occurrence of the KEL*04/KEL*04 genotype. The PCR-SSP genotyping results obtained in this study were accurate as demonstrated by the absence of cross-amplification between the two primer sets used to identify the KEL*03 and KEL*04 alleles. Moreover, the results of the PCR-SSP repeatability testing on 100 randomly selected samples from 400 known genotypes by PCR-SSP and DNA sequencing were consistent with the first round of testing. Genotyping techniques such as PCR-SSP are more applicable and cost-effective than serological techniques for determining the prevalence of antigens in extended blood group systems. While reference laboratories do have high-throughput Luminex-based genotyping technologies available, they require expensive reagents and complicated equipment. The developed PCR-SSP method makes it more affordable to screen a large number of samples for Kp^a^ prediction.

Using the two-tailed Fisher’s exact test with an uncorrected *p*-value of 0.05, there were significant differences in KEL*03 and KEL*04 allele frequencies between individuals in Thai populations and those in South Asian, Japanese, Native American, Alaska Native/Aleut, South African, Brazilian, Swiss, and German populations.^9,11,15,16,17^ However, using the Bonferroni-adjusted *p*-value cutoff of 0.004, the significant differences observed between the Thai populations and the South Asian and Alaska Native/Aleut populations were considered false positives. Therefore, there were only significant differences in KEL*03 and KEL*04 allele frequencies between Thai donors and donors from Japanese, Native American, South African, Brazilian, Swiss, and German populations.^9,11,15,16,17^ This means that anti-Kp^a^ antibodies may be more common among patients in these populations than among Thai patients. The majority (approximately 95%) of Kp(a−b+) donors in Japanese, Native American, Brazilian, Swiss, and German populations would be easily selected to transfuse among patients producing anti-Kp^a^. This is because the Kp(a+) antigen is a low-frequency antigen, and the probability of finding compatible blood units is high.^1,2^ In contrast, only a few donors with the Kp(a+b−) phenotype can serve as donors to patients with the Kp(a+b−) phenotype who have anti-Kp^b^ antibodies.^2^ In this study, the prevalence of the predicted Kp(a−b+) phenotype was similar among Thai donors and individuals from Filipino, Southeast Asian, Chinese, Korean, South Asian, Alaska Native/Aleut, and Hawaiian/Pacific Islander populations.^11^ This finding is consistent with existing data on Kp^a^ alloimmunisation:^1,2^ so far, anti-Kp^a^ has not been reported in the aforementioned populations. Given the low prevalence of Kp(a+b−) observed in this study, autologous donation or compatible blood recruitment from family members will be necessary to provide safe blood transfusion in Thai patients with anti-Kp^b^ antibodies.

A related study on genetic distance analysis of Thai blood donors based on Rh, MNS, Kidd Diego, and Kell blood group genotypes including 10 alleles – RHCE*E, RHCE*e, GYPB*03, GYPB*04, JK*01, JK*02, DI*01, DI*02, KEL*01, and KEL*02 – revealed a close cluster pattern formed by highly related populations, such as those from Thai, Filipino, Southeast Asian, Chinese, and Hawaiian/Pacific Islander populations.^20^ In our study, however, the KEL*03 and KEL*04 frequencies among the Thai donors were similar to those observed in the Filipino, Southeast Asian, Chinese, Korean, South Asian, Hawaiian/Pacific Islander, and Alaska Native/Aleut populations. A large proportion of the current Thai, Filipino, Southeast Asian, Chinese, Korean, South Asian, and Hawaiian/Pacific Islanders populations possibly descended from a common ancestor.^20^ However, the observed differentiation of blood group polymorphisms may have arisen a long time ago in different sub-populations, and there may not have been any significant genetic admixture thereafter. A prior study conducted in Thailand in 2023 to investigate the distribution of the 10 blood group alleles revealed that the Alaska Native/Aleut population differed from the Thai population with regard to pairwise genetic distances.^20^ This differentiation may have been influenced by a combination of cultural practices, particularly within-group marriages, and the consequences of group isolation.^21^ Nevertheless, more research using larger sample sizes on additional relevant blood group alleles may support the findings of genetic similarities within populations and can be used in anthropological studies.

### Limitations

One limiting factor of the PCR-SSP assay stems from the need for samples with known and relatively rare phenotypes, particularly Kp(a+b−) and Kp(a+b+), to ensure the test’s validity and reliability. In addition, an erroneous prediction of the K_0_, Kmod, KEL: 1 weak, and other variants of the *KEL* gene – which are exceedingly rare in Thai and other populations – may occur when using PCR-SSP to determine KEL*03 and KEL*04 alleles. The confirmation of these phenotypes using serological techniques and *KEL* gene sequencing is thus suggested.^2,22^ To obtain a more representative picture of the prevalent *KEL* gene variants among Thai blood donors, the genotypic results reported in this study need to be verified in large-scale studies involving all regions in Thailand.

### Conclusion

This study found a 100% KEL*04 allele frequency in three Thai populations. This data could provide information on KEL*03 and KEL*04 allele frequencies to estimate the risk of alloimmunisation in Thai populations.
